# The Role of the Nuclear Factor-Kappa B (NF-κB) Pathway in SARS-CoV-2 Infection

**DOI:** 10.3390/pathogens13020164

**Published:** 2024-02-12

**Authors:** Periyanaina Kesika, Subramanian Thangaleela, Natarajan Sisubalan, Arumugam Radha, Bhagavathi Sundaram Sivamaruthi, Chaiyavat Chaiyasut

**Affiliations:** 1 Office of Research Administration, Chiang Mai University, Chiang Mai 50200, Thailand; kesika.p@cmu.ac.th (P.K.); sisubalan.n@cmu.ac.th (N.S.); 2 Innovation Center for Holistic Health, Nutraceuticals, and Cosmeceuticals, Faculty of Pharmacy, Chiang Mai University, Chiang Mai 50200, Thailand; 3 Institute of Biotechnology, Department of Medical Biotechnology and Integrative Physiology, Saveetha School of Engineering, Saveetha Institute of Medical and Technical Sciences, Chennai 602105, Tamil Nadu, India; 4 Department of Animal Science, School of Life Sciences, Bharathidasan University, Tiruchirappalli 620024, Tamil Nadu, India

**Keywords:** COVID-19, SARS-CoV-2, NF-κB, inflammation, cytokines

## Abstract

COVID-19 is a global health threat caused by severe acute respiratory syndrome coronavirus 2 (SARS-CoV-2) and is associated with a significant increase in morbidity and mortality. The present review discusses nuclear factor-kappa B (NF-κB) activation and its potential therapeutical role in treating COVID-19. COVID-19 pathogenesis, the major NF-κB pathways, and the involvement of NF-κB in SARS-CoV-2 have been detailed. Specifically, NF-κB activation and its impact on managing COVID-19 has been discussed. As a central player in the immune and inflammatory responses, modulating NF-κB activation could offer a strategic avenue for managing SARS-CoV-2 infection. Understanding the NF-κB pathway’s role could aid in developing treatments against SARS-CoV-2. Further investigations into the intricacies of NF-κB activation are required to reveal effective therapeutic strategies for managing and combating the SARS-CoV-2 infection and COVID-19.

## 1. Introduction

COVID-19, caused by severe acute respiratory syndrome coronavirus 2 (SARS-CoV-2), is a massive health threat all over the globe this century [1,2], resulting in more morbidities and mortalities. Similarly to the flu virus, COVID-19 is also transmitted through microdroplets when sneezing, coughing, or speaking with the infected person without social distancing. SARS-CoV-2 enters the host through the nasal barrier [3]. The COVID-19 outbreak occurred similarly to pneumonia in December 2019 in Wuhan, China. Soon, the pneumonia-like disease rapidly spread and caused an emergency all over the world. The outbreak was officially announced as a global pandemic in January 2020, and the causative agent is SARS-CoV-2 [4,5]. The International Committee on Taxonomy named the virus SARS-CoV-2; the disease was named COVID-19 by the World Health Organisation (WHO) [6].

The infection and activation of the virus can result in a cytokine storm through the release of various chemokines and cytokines. Releasing cytokines can cause an inflammatory response that affects the lungs [7]. The COVID-19 outburst is still considered a potent threat to public health. Many research groups started to evaluate the clinical background of COVID-19 infection. The research found that nuclear factor (NF)-kappa B (NF-κB)-driven inflammatory responses [8,9] are associated with COVID-19. As a result of immune system dysregulation after COVID-19, the unlimited release of proinflammatory cytokines, elevated cytokine levels, and chemokine circulation cause hemorrhage, thrombocytopenia, and systemic inflammation [10].

NF-κB has been an inducible transcriptional model due to its extensive pathophysiological impacts and therapeutical applications [11]. Despite the multifaceted functions of NF-κB, several studies have affirmed that NF-κB operates in conjunction with other signaling pathways and orchestrates diverse responses [12,13]. Inflammatory cytokines, oxidative stress, and infections can activate NF-kB [13]. The triggered NF-κB engages in diverse cellular signaling pathways that influence cell differentiation, proliferation, survival, intercellular communication, and immunomodulation [14,15]. Any abnormalities in NF-κB function further lead to inflammatory and autoimmune conditions, metabolic disorders, and cancer [16,17,18]. This review highlights SARS-CoV-2 infection, NF-κB activation, and its potential therapeutical role in treating COVID-19.

## 2. COVID-19 Infection and Pathogenesis

The zoonotic, positive-sense, single-stranded RNA SARS-CoV-2 virus belongs to the family Coronaviridae in the genus *Betacoronavirus*, subgenus Sarbecovirus [19]. CoV-2 is an enveloped β virus covered with spike glycoproteins and other membrane proteins and showed 80% genetic similarity to CoV-1 and 96.2% to bat CoV RaTG13 [20]. To date, seven human coronaviruses (HCoV) have been found to cause infection in humans, which include alpha coronaviruses (α-CoVs) such as HCov-229E and HCoV-NL63, and beta-coronaviruses (β-CoVs) such as HCoV-HKU1, HCoV-OC43, SARS-CoV, MERS (Middle East respiratory syndrome)-CoV, and SARS-CoV-2 [21].

From observing the past few decades, the epidemics caused by viruses have probably originated from wild animal reservoirs like bats and civet cats, which are then transferred to humans through zoonotic events [22]. In the previous two decennials, three COVID outbreaks have been instigated worldwide. CoVs generally cause lower respiratory tract infection and pathogenicity, resulting in high fatality. SARS-CoV-2 exhibited mild symptoms and severe lung injury and death. HCoV infects humans and causes various clinical conditions, including coughs, moderate febrility, septic shock, progressive respiratory epithelial damage, acute respiratory distress syndrome (ARDS), severe pneumonia, and multiple organ failure in some cases [23,24]. A cohort study of COVID-19 patients showed that enhanced transcriptional activation of NF-κB produces excessive cytokines, tumor necrosis factor-α (TNF-α), and interleukin-6 (IL-6), which in turn cause severe COVID-19 morbidity [8].

The genome of the SARS-CoV-2 virus encodes around 16 non-structural proteins (NSP), which are responsible for mass viral replication, and four structural proteins (the envelope, nucleocapsid, membrane, and spike proteins) that produce new virions and other accessory proteins, namely ORF3a, ORF3b, ORF6, ORF7a, ORF8, ORF9b, ORF9c, and ORF10, which are responsible for viral pathogenesis [25,26,27]. Even though the SARS-CoV-2 virus is genetically similar to SARS-CoV-1, several characteristics, such as variabilities in surface proteins and kinetics of viral loads concerning increased transmission rate, differ between SARS-CoV-1 and -2. In SARS-CoV-2 infection, the viral load peaked at the onset of symptoms. It then reduces further, which indicates that CoV2 infectiousness is crucial during the initial first five days after the onset of symptoms [28].

The experiments conducted in vitro on the spike proteins of the virus suggested a potential affinity of the protein to ACE2 [29]. The SARS-CoV-2 virus has a high affinity towards the upper respiratory tract and conjunctiva [30]. SARS-CoV-2 and ACE2 binding results in enhanced transmissibility of viral particles and severity of infection in humans [31,32]. As with SARS-CoV-2, human CoV-LN63 and SARS-CoV-1 bind to the ACE2 receptor but the severity of the disease differs, which suggests that the potential for pathogenicity of other important virulent factors might differ between these three coronaviruses [33]. Other receptors such as neuropilin 1 and proteases such as cathepsin L, TMPRSS11D, and TMPRSS13 also favor SARS-CoV-2 entry into human cells [34,35,36].

In addition to lung injury, extra-pulmonary disturbances were observed in patients [37]. SARS-CoV-2 RNA was detected in several other organs, such as the kidney, colon, spleen, skin, heart, and brain [38,39,40], which indicates that those cells might express ACE2 receptors and support the binding of viral particles [38,41].

The ACE2-RBD of SARS-CoV2 is identical to the RBD of SARS-CoV-1, but SARS-CoV-2 has more specificity and 10 to 20 times higher binding affinity towards ACE2 [42]. The intricate configurations of human angiotensin-converting enzyme 2 (hACE2) in conjunction with the RBDs of SARS-CoV-2 variants, namely BA.1.1, BA.2, and BA.3, elucidate that the elevated hACE2 binding affinity exhibited by BA.2 in comparison to BA.1 is attributable to the absence of the G496S mutation in BA.2. Additionally, the R346K mutation observed in BA.1.1 significantly impacts the interaction network within the BA.1.1 RBD/hACE2 interface, inducing long-range alterations. This mutation contributes to the superior hACE2 affinity of the BA.1.1 RBD compared to the BA.1 RBD [43]. The ACE2 receptor is the main entry point of the virus, thus allowing it to circulate through the bloodstream and initiate a widespread systemic response characterized by hyperinflammation, which has been linked to various inflammatory diseases [44]. Studies conducted among the Chinese population showed that elevated levels of IL-6 and fibrinogen, along with systemic inflammation, are the key indicators associated with poorer diagnoses, ultimately contributing to higher mortality rates [45].

HCoV targets the alveolar epithelial cell type II and disrupts the NF-κB pathway, leading to multiple organ failures and fatality [46]. Alveolar epithelial type II cells, recognized for their pivotal role, are responsible for synthesizing, secretion, and recycling all constituents of pulmonary surfactant, a critical regulator of alveolar surface tension in mammalian lungs [47]. Autopsy studies from COVID-19 patients showed that the pathological changes are similar to those in patients infected with SARS and MERS [48]. Pneumonia, lymphopenia, and respiratory failure are common among COVID-19, SARS, and MERS patients [23,49,50]. SARS-CoV-2 infection produces modest respiratory disease accompanied by coughing, fever, headache, diarrhea, dyspnoea, hypoxemia, and severe respiratory failure [19,51].

Pulmonary vascular leakage, aerated lung tissue loss, and inflammation are the consequences of ARDS. Respiratory failure is accompanied by systemic hyperinflammation, the release of proinflammatory cytokines (IL-1, IL-6, IL-8, and TNF), and other inflammatory markers (Ferritin, C-reactive protein, and D-dimer) [52].

The stages of viral infection were explained as viral invasion, replication, disturbed immune response, and multi-organ damage. Once the viral replication is completed inside the target cell, the viral particles are released into the target cells, such as alveolar epithelial cells, and cause damage to them. During this phase, PAMPs (pathogen-associated molecular patterns) and DAMP (damage-associated molecular pattern) molecules are released and induce infiltration of inflammatory cells, cytokines, chemokines, proteases, and free radicals. The pathological signs (vascular congestion, alveolar damage, intra-alveolar edema, hemorrhage, hyperplasia of pneumocytes, proteinaceous exudate, and patchy inflammatory cellular infiltration) of SARS-CoV-2 are similar to the pathological characteristics of SARS-CoV-1 and MERS-CoV [53,54].

The cytopathic effects of SARS-CoV-2, reduction of ACE2, imbalance of the renin-angiotensin-aldosterone system, and dysregulated immune response result in a cytokine storm, abnormal blood clotting linked to the release of factors promoting blood coagulation, and microvascular thrombosis triggered by virus-induced blood vessel damage, activation of the complement system, and development of autoimmune reactions [55,56,57,58]. The binding of the SARS-CoV-2 spike protein to the ACE2 receptor and the fusion of the viral envelope to the cell membrane mediated by TMPRSS2 induce the first line of the innate immune response. PAMPs are recognized by PRRs (pattern recognition receptors) such as TLRs (Toll-like receptors). Thus, the recognition of these molecules activates transcription factors and IRFs (interferon regulatory factors), resulting in the production of type I interferons (IFNs), chemokines, and proinflammatory cytokines [59].

Activation of the immune system recruits inflammatory myeloid cells, neutrophils, CD8 T cells, and NK (natural killer) cells. The cytotoxic function of CD8 T and NK cells helps clear the virus-infected cells [60]. Any accumulation of virus-infected cells due to the failed or active clearance of infected cells would produce a hyperinflammatory state called macrophage activation syndrome, ultimately resulting in lung damage [61,62]. Immune cell profiling of COVID-19 patients’ blood samples indicated that the diminution of CD4^+^, CD8^+^, T, and NK cells could cause lymphopenia [1,63]. Several cardiovascular conditions, including myocardial injury and infarction, myocarditis, dysrhythmias, cortical venous thromboembolic events, and heart failure, have been observed in COVID-19 patients [64].

## 3. NF-κB Signaling Pathways

**Figure 1:**
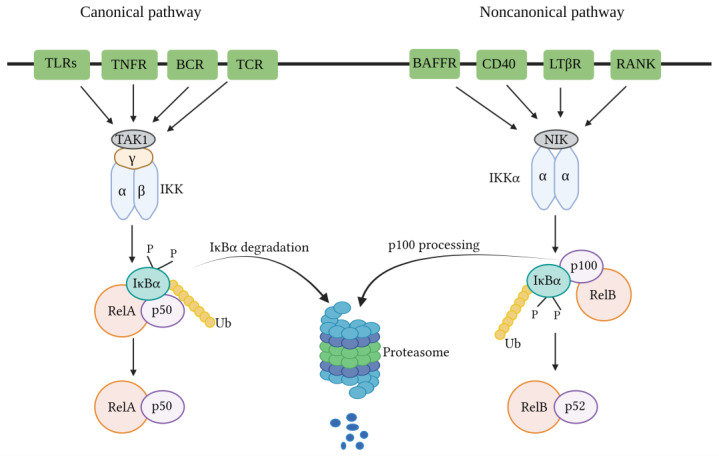
NF-κB signaling (canonical and non-canonical) pathways. Several signals, including signals associated with immune receptors, could trigger the canonical pathways, which involve IKK complex activation by Tak1, IKK-mediated IκBα phosphorylation, and following degradation, consequential transient nuclear translocation of the prototypical NF-κB heterodimer P50/RelA. In the case of the non-canonical pathway, signals from a subset of TNFR members will trigger phosphorylation-induced p100 processing, and this pathway is NIK- and IKKα-dependent, not trimeric IKK complex-dependent, intervening in the persistent activation of the RelB/p52 complex. BAFFR: B-cell-activating factor belonging to TNF family receptor; BCR: B cell receptor; CD40: Cluster of differentiation 40; TLRs: Toll-like receptors; TNFR: Tumor necrosis factor (TNF) receptor; TCR: T cell receptor; LTβR: Lymphotoxin β-receptor; TAK1: IKK-activating kinase-1; IKK: IκB kinase; RANK: Receptor activator for nuclear factor κB; RelA: Protein of mammalian NF-κB family; NIK: NF-κB-inducing kinase (Recreated with permission based on Sun, 2011 [67]).

Typically, NF-κB remains inactive and confined to the cytoplasm in the quiescent state of most normal cells. It associates with a specific inhibitor known as the IκB protein, which interacts with the Rel homology domain (RHD) of NF-κB, disrupting its nuclear localization sequence functionality. The inhibitor proteins, exemplified by IκBα, IκBβ, and IκBγ, are characterized by the presence of six to seven ankyrin repeats. These ankyrin repeats play a crucial role in facilitating the binding of inhibitor proteins to the RHD of NF-κB, thereby disrupting its nuclear localization sequence functionality [65]. The C-terminal shares of the NF-κB2/p100 and NF-κB1/p105 precursors also harbor ankyrin repeats, serving as inhibitors. These IκBs, akin to IκBa, IκBb, and IκBg, play a role in retaining their associated Rel proteins within the cytoplasm. The separation of the NF-κB protein from its inhibitors is necessary to activate NF-κB. Two primary signaling pathways, canonical and non-canonical, govern the dissociation of IκB protein inhibitors from the NF-κB dimer [66] (Figure 1). Several regulatory mechanisms, including site-specific p100 phosphorylation, ubiquitination, sumoylation, and constitutive processing, of NF-κB pathways have been detailed previously [67].

### 3.1. Canonical NF-κB Pathway

The canonical NF-κB pathway governing NF-κB activation is dependent upon the inducible degradation of IκBs, notably IκBα. It concludes in the nuclear translocation of diverse NF-κB complexes, with a predominant occurrence of the P50/RelA dimer [68,69]. The inducible degradation of IκBα is carried out through its phosphorylation, a process catalyzed by the IκB kinase (IKK). The IKK is a trimeric complex consisting of two catalytic subunits, namely IKKα and IKKβ, alongside a regulatory subunit identified as IKKγ, also referred to as the NF-κB essential modulator (NEMO) [70]. In canonical NF-κB signaling cascades, such as those initiated downstream of tumor necrosis factor receptor 1 (TNFR1), IKKβ is essential and adequate for the phosphorylation of IκBα at Ser32 and Ser36, as well as IκBβ at Ser19 and Ser23. IKKα can facilitate IκBα phosphorylation, playing a pivotal role in canonical NF-κB-dependent transcriptional responses. Despite some exceptions, wherein specific instances are detailed below, it is observed that both canonical and non-canonical pathways employ TNF receptor-associated factor (TRAF) family members for activation. Notably, in the canonical pathway, NEMO-dependent signaling leading to typical IκBs, along with the involvement of receptor-interacting proteins (RIPs), is also required [68].

RIPs serve as pivotal adapters in canonical NF-κB signaling. These proteins exhibit dual functionality by acting both upstream of and accompanied by TRAF proteins to activate IKK. In the NF-κB signaling pathways, RIPs function as authentic adapters by engaging with upstream signaling modules through well-characterized protein-binding domains. Furthermore, they facilitate the recruitment of the IKK complex via binding to NEMO. The RIP family members play crucial roles in numerous TRAF-dependent pathways, including those initiated by the TNF receptor superfamily and the Toll/IL-1 receptor.

**Figure 2:**
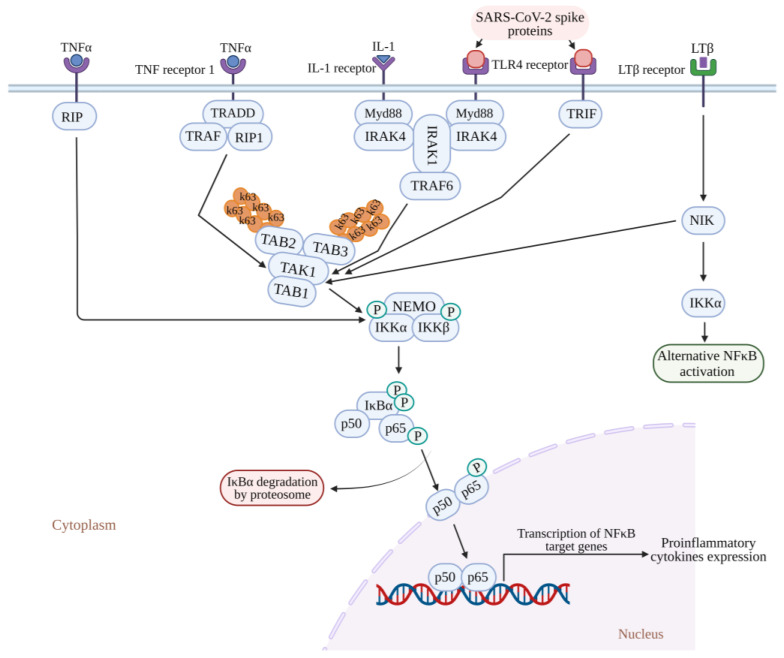
Activation of the NF-kB signaling pathway induced by SARS-Co-V-2 and mediated by RIP/TRAF-dependent pathways or RIP-dependent/TRAF-independent IKKβ activation. The pathways activating NF-κB are RIP/TRAF-dependent pathways and RIP-dependent/TRAF-independent IKKβ activation, which may encompass antigen receptor signaling. Signaling to IKK downstream of RIPs and TRAFs involves several kinases implicated in NF-κB signaling pathways. Specifically, IKK activation is mediated by TAK1 in the TNF-alpha and IL-1 signaling pathways. RIP/TRAP-dependent pathways, including TNF receptor 1, IL-1 receptor, and TLR4 receptor signaling stimulated with their ligands (such as TNF-alpha, IL-1, and SARS-Co-V-2 spike proteins, respectively), recruit the binding of adaptor proteins such as RIP1-TRAF2-TRADD with TNF receptor 1, and MyD88-IRAK1-TRAF6 with the TLR4/IL-1 receptor. SARS-Co-V-2 spike proteins stimulate TLR4 signaling that recruits the binding of adopter protein TRIF, which leads to TAK1-mediated IKK activation. K63-linked polyubiquitin chain formation occurs by catalyzation with E3 ligase TRAF. K63-linked polyubiquitin chains bind to TAB2 and TAB3 subunits, thereby leading to the assembly and activation of the TAK1-TABs complex. IKKs bind to NEMO, forming an IKK-NEMO complex. The TAK1-TABs complex phosphorylates the IKKα and IKKβ present in the IKK-NEMO complex, leading to the activation of IKKs, which further activates transcription factor NF-κB (p65/p50). The transcription of NF-κB target genes leads to the expression of proinflammatory cytokines. LT-beta signaling-mediated alternative NF-κB activation through NIK is independent of TAK1, whereas NIK signaling mediates TAK1-dependent classical NF-κB activation. In RIP-dependent/TRAF-independent IKKβ activation, TNFα stimulates the TNF receptor 1, which induces the binding of adopter protein RIP that further recruits the IKK complex by direct interaction with NEMO and leads to IKKβ activation and NF-κB activation. NF-κB: Nuclear factor kappa B; RIP: Receptor-interacting protein; TRAF: tumor necrosis factor receptor (TNF-R)-associated factor; IKK: IκB kinase; MyD88: myeloid differentiation primary response protein 88; TRADD: TNFR-associated protein with a death domain; IRAK1: IL-1 receptor-associated kinase 1; IRAK4: IL-1 receptor-associated kinase 4; TAK1: Transforming growth factor-beta-activated kinase 1; TAB 1: TAK1-binding protein 1, TAB 2: TAK1-binding protein 2; TAB 3: TAK1-binding protein 3; NEMO: NF-κB essential modulator; TLR4: Toll-like receptor 4; TNF receptor: Tumor necrosis factor receptor; IL1: Interleukin 1; TNFα: Tumor necrosis factor-α; LTβ: Lymphotoxin β; NIK: NF-κB-inducing kinase.

Moreover, RIP family members apply significance without a clear requirement for TRAFs. These pathways, characterized by RIP-dependent and TRAF-independent IKKβ activation, may encompass antigen receptor signaling and responses to DNA damage [68]. Signaling to IKK downstream of RIPs and TRAFs involves several kinases implicated in NF-κB signaling pathways. Specifically, in canonical NF-κB pathways, this function is predominantly executed by TGFβ-activated kinase-1 (TAK1) [71,72]. Hence, a more suitable categorization of NF-κB pathways involves classification as canonical or non-canonical based on the necessity for NEMO or the IκB protein subject to phosphorylation and degradation/processing. Specifically, the canonical pathway involves IκBα, IκBβ, and IκBε, while the non-canonical pathway involves p100, not relying on the requirement for IKKα or IKKβ [68] (Figure 2).

### 3.2. Non-Canonical NF-κB Pathway

The non-canonical NF-κB signaling pathway is responsible for the activation of the p52/RelB NF-κB complex through a distinctive mechanism predicated upon the inducible processing of p100 instead of the conventional degradation of IκBα [67]. Beyond its role as the precursor of p52, p100 serves a dual function similar to an IκB-like molecule, applying a preferential inhibitory effect on the nuclear translocation of RelB [73]. At the core of the non-canonical pathway lies NF-κB-inducing kinase (NIK), a pivotal signaling component that assimilates signals from a subset of TNF receptor family members. NIK, in turn, initiates the activation of IκB kinase-α (IKKα), a downstream kinase responsible for initiating the phosphorylation and subsequent processing of p100 [67]. NIK is the initial element discovered in the non-canonical NF-κB signaling pathway [74]. NIK, categorized as a MAP kinase kinase kinase (MAP3K), was initially implicated in the activation of NF-κB through the TNF receptor (TNFR) pathway [75]. NIK applies its functional role by activating a subsequent kinase, IKKα [76]. In contrast to IKKβ and IKKγ, pivotal constituents of the canonical NF-κB pathway, IKKα specifically, and not IKKβ or IKKγ, are essential for the execution of non-canonical NF-κB signaling [76,77,78,79].

The recruitment of NIK by receptors has the potential to elevate its local concentration, thereby potentially inducing autophosphorylation without necessitating an increase in NIK expression. This mechanism of NIK activation may be operative during the initial stages of non-canonical NF-κB signaling, suggesting a probable scenario wherein NIK accumulation could play a role in sustaining non-canonical NF-κB signaling. Nevertheless, an alternative perspective suggests that the activation of NIK by specific receptor signals, especially within distinct cell types, may exclusively rely upon the early-phase mechanism [67]. Genetic evidence indicates that this NF-κB pathway governs critical biological processes, including lymphoid organogenesis, the survival and maturation of B cells, activation of dendritic cells, and the regulation of bone metabolism [78]. Furthermore, deregulated non-canonical NF-κB signaling is linked to lymphoid malignancies [67]. Signal-induced non-canonical NF-κB signaling is characterized by sustained degradation of the T3-T2-cIAP E3 (refers to a specific set of proteins such as T3 (TRAF3), T2 (TRAF2), cIAP (cellular Inhibitor of Apoptosis) involved in the regulation of NF-κB signaling) components, specifically TRAF3 and/or TRAF2 [80]. Notably, the accumulation of NIK is arrested early in this process and remains consistently maintained at a steady level throughout non-canonical NF-κB signaling [80]. This phenomenon is primarily attributed to a feedback mechanism governing the regulation of NIK, facilitated by its downstream kinase, IKKα [80].

## 4. NF-κB Signaling in COVID-19 Infection

Generally, the NF-κB transcription factor regulates immune cell functions and gene expression to various pathogenic stimuli. Thus, the proinflammatory stimuli induce the NF-κB signaling cascade. In the case of COVID-19 infection, the abnormal NF-κB activation results in elevated levels of immune cells and cytokine storm. The activation and strong involvement of NF-κB during COVID-19 infection show that NF-κB might be a potential target [81]. COVID-19 patients displayed elevated levels of IL-1β, IL-6, and TNF [82,83]. Such an enhanced cytokine storm induces ARDS and high mortality rates among COVID-19 patients [84]. The higher secretion of cytokines can also enhance other viral infections, including hantavirus pulmonary syndrome, H5N1, influenza virus, and SARS-CoV-1 [85,86,87,88], and produce a great impact over the negative feedback control of the immune response, resulting in the continuous cytokine secretion that perpetuates the positive feedback response of immune cells. Recruiting even more immune cells towards the infection site causes enhanced inflammation, COVID-19 disease progression, multiple organ failures, and fatality [89]. The function of NF-κB is modulated by an IκB (inhibitor of NF-κB) family of proteins such as IκBα, β, γ, and ε, and Bcl-3 [90,91]. Highly conserved NF-κB dimeric transcription factors include Rel-like domain-containing proteins RelA, RelB, RelC, p105, and p100. P105 and p100 are latent forms of NF-κB proteolytically cleaved to generate active forms, namely p50 and p52 [90]. RelA and p50 form heterodimers and act as major NF-κB complexes in most cells. Studies have shown that NF-κB regulates 400 human genes that code for cytokines, chemokines, and genes responsible for stress response, cell growth, and cell death [92].

Basal expression of NF-κB is observed in cells under pathological circumstances, including exposure to pathogenic stimuli, stress hormones, radiation, and free radicals [93]. The phosphorylation and subsequent degradation of IκBβ result in the release of p50 and p52 subunits, which then translocate into the nucleus. Within the nucleus, these subunits serve as putative transcription factors, governing the transcription of genes essential for the inflammatory response [94,95]. In addition to various other cellular and immunological functions [90,93,96], NF-κB signaling is vital for secondary lymphoid organ development, as well as immune cell production and activation upon inflammation and microbial invasion [90,93,97]. Pathogenic stimulation of the immune system causes inflammation, resulting in the production of cytokines, interleukins, and microbial toxins (lipopolysaccharides), which in turn induce a NF-κB cascade mediated by interferon-γ signal transducers, transcription activators, and TGF-β [98,99].

Viral infections trigger the host’s innate immune system, which recognizes the viral particles and products, including nucleic acids, capsids, and other proteins. It induces a defense mechanism by initiating an NF-κB signal, attenuating viral infection [92]. The upregulation of NF-κB-mediated inflammasomes (receptors/sensors of the innate immune system) modulating the activation of caspase-1 and responsible for eliciting an inflammatory response in reaction to infectious microorganisms and molecules originating from host proteins [100] following viral invasion and infection has been documented [101]. Consequently, activated NF-κB signaling leads to the synthesis of proinflammatory factors, its activators, oxidative stress, and cellular apoptosis across various tissues [102]. Investigations into the outbreaks of SARS-CoV and SARS-CoV-2 have centered on the abnormal activation of NF-κB signaling facilitated by cytokine storms and resultant tissue damage [103,104,105]. These inquiries underscore the potential of NF-κB as a promising target in combatting SARS-CoV infections.

**Figure 3:**
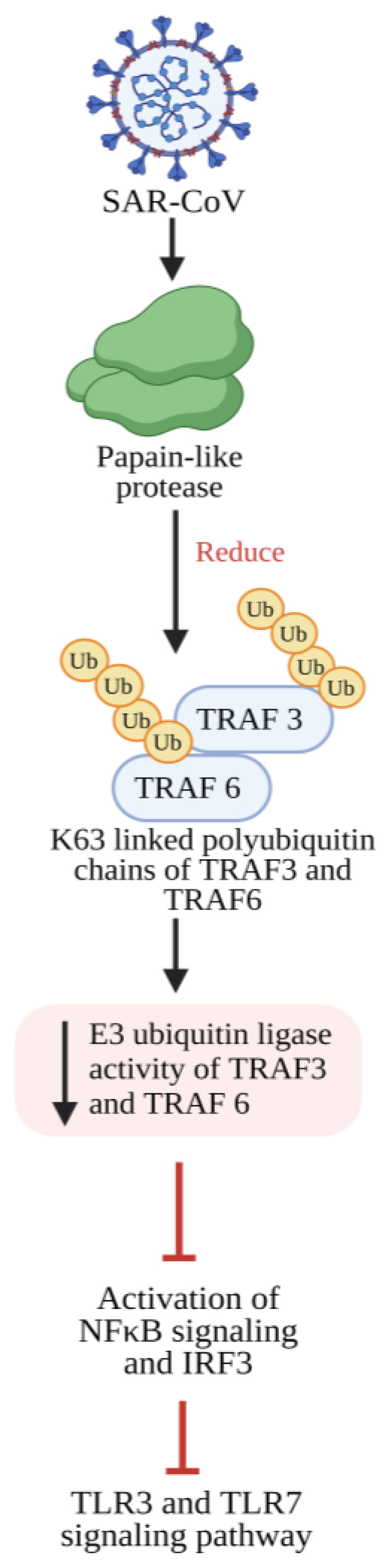
SARS-CoV-2 papain-like protease inhibits the TLR3 and TLR7 signaling pathway-mediated inflammatory and antiviral response. Papain-like protease decreases the Lysine 63 (K63)-linked polyubiquitin chains of TRAF3 and TRAF6, which leads to reduced E3 ubiquitin ligase activity from TRAF3 and TRAF6, thereby preventing the activation of IRF-3 and NF-κB and further inhibiting TLR3- and TLR7-mediated expression of INF1 and proinflammatory cytokines. TLR3: Toll-like receptor 3; TLR7: Toll-like receptor 7; NF-κB: Nuclear factor kappa B; IRF-3: Interferon regulatory transcription factor 3; TRAF3: tumor necrosis factor receptor (TNF-R)-associated factor 3; TRAF6: tumor necrosis factor receptor (TNF-R)-associated factor 6; INF1: Interferon 1.

Viruses evolved with different infection strategies, including targeting ubiquitination to modulate NF-κB signaling by evading host surveillance. The viral genome encoding a viral E3 ligase targets components of the NF-κB proteasome, inducing proteasomal degradation and consequently suppressing NF-κB signaling. Few viruses encode a viral deubiquitinating enzyme to remove the K63-linked polyubiquitin chain and prevent NF-κB activation. In the case of SARS-CoV, the papain-like protease relieves the K63-linked polyubiquitin chain of TRAF3 and TRAF6 and blocks the activation of interferon-regulatory factor-3 and NF-κB [106,107,108] (Figure 3).

**Figure 4:**
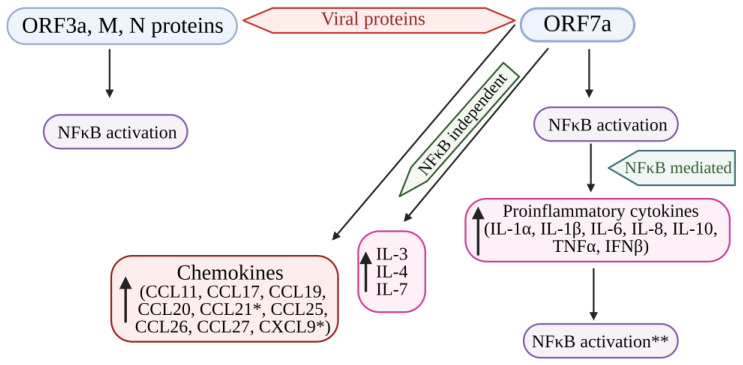
Cytokine storm induced by SARS-CoV-2 proteins (ORF7a, ORF3A, M, and N proteins) mediated by the activation of NF-kB. The structural viral proteins (M and N proteins) and predicted accessory viral proteins (ORF3a and ORF7a proteins) activate the NFκB signaling pathway. Among the 4 viral proteins, ORF7a is the most potent activator of proinflammatory cytokines, whose expression is mediated by NFκB activation. Cytokines such as IL-3, IL-4, and IL-7 were also upregulated by ORF7a. In addition, ORF7a promotes the expression of chemokines. The over-expression of cytokines further activates the NFκB pathway, resulting in the SARS-CoV-induced cytokine storm. * Significantly elevated in COVID-19 patients. ** Overexpression of cytokines further enhances NFκB activation. NFκB: Nuclear factor kappa B; ORF7a: Open-reading frame 7a; ORF3a: Open-reading frame 3a; M: Membrane protein; N: Nucleocapsid protein; IL: Interleukin; TNFα: Tumor necrosis factor-α; IFNβ: Interferon-β; CCL11, 17, 19, 20, 21, 25, 26, 27: Eosinophil chemotactic factors; CXCL9: Chemokine; ↑: Increased or upregulated.

NF-κB encodes many genes that function as an antiviral defense system. Viral invasion and infection activate the NF-κB transcription factors. Viruses with their key components evade the NF-κB antiviral responses and substantiate the viral infection [109]. Su et al. [89] evidenced that the ORF3a, ORF7a, M, and N proteins of SARS-CoV-2 activate NF-κB signaling. Among these four viral proteins, ORF7a induces the NF-κB-regulating proinflammatory cytokines. In addition, some of the cytokines (IL-3, IL-4, IL-7, and IL-23) and chemokines, including CCL11, CCL17, CCL19 to 22, CCL25 to 27, and CCL29, were upregulated in COVID-19 patients, indicating that SARS-CoV-2 modulates NF-κB signaling and the expression of inflammatory cytokines. Hence, the protein ORF7a might be targeted to develop a COVID-19 treatment strategy by controlling the uncontrolled inflammation in COVID-19 patients [89] (Figure 4).

Viruses activate the NF-kB signaling cascade to promote infection; viruses interact with NF-κB signaling components, especially PRRs and IκB kinase [94,110]. Certain viruses, including herpes simplex virus-1, human immunodeficiency virus, and bovine foamy virus, promote infection and viral gene expression using NF-κB transcription factors [110,111,112,113]. SARS-CoV-2 infection induces an imbalance in the inflammatory responses through weak production of IFNs and hyperproduction of proinflammatory cytokines, leading to ARDS [98,114]. Several clinical studies have postulated that cytokines (IL-1, IL-6, IL-8, and TNF-α) and chemokines were abnormally raised to high levels in COVID-19 patients [23,82,115,116]. NF-κB governs the manifestation of proinflammatory cytokines, and through a positive feedback mechanism, these cytokines further enhance NF-κB activity [117]. In COVID-19 patients, increased levels of proinflammatory cytokines intensify NF-κB activation [115]. The upregulation of NF-κB likely promotes the inflammatory response in COVID-19 patients. The viral proteins responsible for immune activation and the exact molecular mechanisms behind such activation have remained unidentified.

Su et al. identified four SARS-CoV-2 proteins as activators of NF-κB. Among these, ORF7a emerged as the most potent inducer of NF-κB and, consequently, a producer of proinflammatory cytokines. Patients who succumbed to COVID-19 exhibited significantly elevated chemokines and T cell recruitment and survival, such as CXCL9 and CCL21. The ORF7a protein also significantly elevates CXCL9 and CCL21 expression. This shows the crucial role of ORF7a in manipulating cytokine and chemokine responses during SARS-CoV-2 infection, and further studies concerning ORF7a are required to unravel the molecular basis for the activation of cytokines [89].

SARS-CoV-2 induces a substantial increase in proinflammatory cytokine production (Figure 4). Previous research on SARS-CoV-1’s ORF3a, M, ORF7a, and N proteins demonstrated the boost in NF-κB activity [103,118,119]. The papain-like protease plays a crucial role in viral replication by cleaving a specific site within the viral replicase and eliminating ubiquitin from cellular proteins. In SARS-CoV, the papain-like protease removes the Lys63-linked ubiquitin chains from TRAF3 and TRAF6 [106]. Papain-like protease disrupts the polyubiquitination process of IκBα and prevents the activation of NF-κB [120] (Figure 3). Persistent activation of NF-κB in individuals with established metabolic syndromes elevates their susceptibility to cardiac complications; with coupled cytokine activation, this condition can result in cardiac injury [64].

### Recent Knowledge of the Role of NF-kB in COVID-19

In recent investigations, the role of NF-kB in COVID-19 has garnered significant attention, shedding light on its potential implications in the pathogenesis and immune response to viral infection. NSP14 mutants were employed, and cells with knockouts (KOs) in host factors within the NF-κB signaling pathways were generated to elucidate the molecular mechanism underlying NSP14-induced NF-κB activation. It demonstrated that full-length NSP14 necessitates methyltransferase (MTase) activity for effective NF-κB induction. Specifically, the wild-type NSP14 (NSP14 WT), as opposed to an MTase-defective mutant, exhibits low expression levels, and its inherent post-translational instability is mediated through proteasomal degradation. Furthermore, the binding of SARS-CoV-2 NSP10 or the addition of the co-factor S-adenosylmethionine stabilizes NSP14, enhancing its potential to activate NF-κB [121]. The NSP14’s stimulation of canonical NF-κB activation relies on NF-κB factor p65/RelA downstream of the NEMO/IKK complex. In contrast, RelC or non-canonical RelB is dispensable for inducing NF-κB transcriptional activity. Notably, NSP14 overexpression fails to induce canonical IκB kinase β (IKKβ)/NF-κB signaling. Co-immunoprecipitation assays do not detect stable associations between NSP14 and NEMO or p65, suggesting that NSP14 indirectly activates NF-κB through its methyltransferase activity. The data provide a comprehensive framework elucidating how NSP14 can enhance basal NF-κB activation, potentially contributing to elevated cytokine expression in SARS-CoV-2-infected cells [121].

Similarly, in another study, the initial NF-kB cluster exhibits a myeloid-centric profile comprising 904 Transcriptional Regulatory Sequences (TSRs) that are co-enriched for motifs associated with CCAAT/enhancer-binding protein (CEBP) and Spi-1 Proto-Oncogene (PU.1) proteins, recognized as Lineage Determining Transcription Factors (LDTFs) for the myeloid compartment. This myeloid NF-kB cluster demonstrates a robust positive correlation with severe lung injury and significantly overlaps the open chromatin regions of neutrophils [122]. In contrast, the second NF-kB TSR cluster is lymphocyte-centric and negatively correlates with the lung injury score. The lymphoid NF-kB cluster demonstrates co-enrichment for the HeLa E-box binding protein (HEB) motif, a critical E-protein in T cell development [123]. Notably, the level of enrichment for the CEBP and PU.1 motif in the lymphoid NF-kB cluster is comparatively lower than in the myeloid cluster [122].

The public genome-wide NF-kB p65/RelA binding data from Chromatin Immunoprecipitation Sequencing (ChIP-seq) were analysed [124,125]. The TSRs within the myeloid NF-kB cluster exhibited substantial overlap with the NF-kB ChIP-seq signal derived from activated monocyte-derived macrophages. Conversely, the lymphoid NF-kB cluster significantly overlapped with the NF-kB ChIP-seq signal derived from activated CD4^+^ T cells. These findings underscore the utility of motif analysis applied to co-regulated TSRs in providing comprehensive insights into activated pathways, the specific transcription factors (TFs) governing them, and the distinct cell types wherein these regulatory processes are active [122].

Similarly, the myeloid NF-kB cluster demonstrates enrichment for the Recombining Binding Protein Suppressor of Hairless (RBPJ) motif, which serves as the transcriptional regulator of the Notch signaling pathway. This regulator interacts with NF-kB in various inflammatory contexts [122,126,127]. Understanding these interrelationships allows therapeutic potential, as targeting individual TFs may impact multiple pathways. In contrast, targeting cooperative TFs may offer pathway specificity while mitigating potential off-target effects [122,128].

Given the pronounced correlation between the identified transcriptional signatures, a subsequent network analysis was conducted on the top 50 genes of Component 187 to elucidate potential biological mechanisms underlying the identified IL2-AIS. The analysis indicated a central involvement of the transcription factor NF-κB in regulating this transcriptional program. It is supported by the presence of several target genes previously documented to be modulated by NF-κB in SARS-CoV-2-infected CD8^+^ T cells, including NFKBIA, NFKBIZ, TNFAIP3, and CXCR4 [129]. A distinctive reversal in the regulation pattern of the core genes within this NF-κB-regulated transcriptional network was observed in COVID-19 compared to low-dose IL-2 treatment in type 1 diabetes. Importantly, all identified NF-κB target genes exhibited positive loading scores for Component 187, indicating an elevated expression of early response factors such as NF-κB and the transcription factor complex AP-1. It suggests the identified NF-κB target genes sustain the proinflammatory gene expression profile in circulating immune cells during the post-acute phase of COVID-19 [130].

A significant increase in the expression levels of NF-κB and NF-κB-regulated proinflammatory genes was demonstrated in association with SARS-CoV-2 infection in Calu-3 cells. Sulforaphane (SFN) inhibits SARS-CoV-2 replication, as evidenced by the analysis of intracellular N-protein sequences or the released virus [131]. SFN inhibits the upregulation of NF-κB expression in SARS-CoV-2-infected cells. Notably, molecular docking studies suggested a direct interaction of SFN with the DNA binding region of NF-κB. Further investigations into the inhibitory effects of SFN on the expression levels of IL-1β and IL-8, representative of proinflammatory genes implicated in the COVID-19 cytokine storm, should be undertaken to determine whether the observed SFN-mediated inhibitory effects extend to other genes associated with the hyper-inflammatory state observed in COVID-19 [131].

The peripheral blood mononuclear cells (PBMCs) from controls and COVID-19 patients and cells from corresponding broncho-alveolar lavage fluid (BALF) were systematically profiled. The result revealed a reduction in dendritic cells (DCs) and an augmentation of monocytes reminiscent of myeloid-derived suppressor cells (MDSCs), which are strongly associated with lymphopenia and inflammatory manifestations in the circulatory system of severe COVID-19 patients. Importantly, these MDSC-like monocytes exhibited a state of immune paralysis [132]. Conversely, monocyte-macrophages within the BALFs of COVID-19 patients exhibited enhanced production of myriad cytokines and chemokines, accompanied by a diminished secretion of interferons. Severe COVID-19 patients had significantly reduced frequencies of peripheral T cells and natural killer (NK) cells, particularly within innate-like T cell populations and various CD8^+^ T cell subsets, when contrasted with their healthy counterparts.

Interestingly, activated CD4^+^ T cell subsets, including Th1, Th2, and Th17-like cells, demonstrated heightened proportions and greater clonal expansion in severe COVID-19 patients. Notably, patients’ peripheral T cells showed no indications of exhaustion or heightened cell death. However, T cells isolated from BALFs exhibited elevated levels of key cytokines such as IFNG, TNF, CCL4, and CCL5. Paired T-cell receptor (TCR) tracking illuminated a substantial recruitment of peripheral T cells to the lungs of severe COVID-19 patients [132].

The examination of viral gene expression features and evolutionary distinctions in SARS-CoV-2-infected cells from the BALF of patients experiencing moderate and severe COVID-19 was conducted using single-cell and bulk tissue transcriptome data. Detectable SARS-CoV-2 sequences were identified in eight types of immune-related cells, including macrophages, T cells, and NK cells. The differential expression of the SARS-CoV-2 ORF10 gene in severe versus moderate samples was observed. Specifically, the ORF10 gene exhibited abundant expression in the infected cells of severe cases, whereas its expression was scarcely detectable in the infected cells of moderate cases. Consequently, the ORF10 to nucleocapsid (N) expression ratio was significantly higher in severe cases than in moderate cases [133].

Additionally, TRSs of viral leader sequence-independent fusions with a 5′ joint point at position 1073 of the SARS-CoV-2 genome were predominantly detected in patients with fatal outcomes, suggesting its potential as an indicator of clinical prognosis. The analysis of motifs in the TRS of viral leader sequence-dependent fusion events of SARS-CoV-2 was compared with those in SARS-CoV, shedding light on their evolutionary trajectory. The findings underscore the potential roles and predictive features of viral transcripts in the pathogenesis of COVID-19 in moderate and severe cases [133].

## 5. NF-κB Pathway: A Pharmacological Target in COVID-19

Targeting the NF-κB pathway in cellular defense is crucial in COVID-19. The NF-κB pathway plays a vital role in defense against infections. It helps control important genes that create proteins to fight viruses and other harmful invaders. In COVID-19, this pathway can become active and cause excessive inflammation, which can be harmful. Scientists are looking into ways to regulate this pathway using drugs to help control the immunological response in COVID-19 patients. By targeting the NF-κB pathway, researchers aim to find treatments to help balance the immune system’s response and improve outcomes for people with COVID-19 [134].

### 5.1. Inflammatory Response Triggered by the SARS-CoV Spike Protein

The study found that immune cells (macrophages) release the inflammatory proteins (IL-6 and TNF-α) while exposed to the spike protein of SARS-CoV. The release of IL-6 and TNFα depends on the spike protein quantity and exposure duration, with the breakdown of IκBα playing a crucial role in NF-κB activation. Inhibiting this step reduces inflammatory protein release, highlighting NF-κB as a key signaling pathway for the spike protein-induced inflammatory response in SARS-CoV [135].

Drugs constraining NF-κB activation, such as caffeic acid phenethyl ester, Bay 11–7082, and parthenolide, lowered inflammation by reducing the production of inflammatory molecules in mice infected with SARS-CoV. Furthermore, blocking NF-κB helped protect against lung damage and improved the survival of the mice after SARS-CoV infection [104]. Research reported that during the SARS-CoV and MERS-CoV outbreaks, viral proteins such as NSP1, NSP3a, NSP7a, spike proteins, and nucleocapsid proteins activated the NF-κB pathway, thereby increasing disease severity and fatality [90,103,104]. For instance, nsp3 of SARS-CoV-2 is characterized by the presence of the SARS-unique domain (SUD) and the papain-like protease (PLpro). These elements collectively counteract the antiviral effects exerted by the p53 protein. Experimental evidence from in vitro and in vivo studies suggests that the combined action of SUD and PLpro results in the degradation of p53, thereby inhibiting the host cell’s defense response. Notably, this inhibition is achieved through stabilizing the E3 ubiquitin ligase RCHY1, indicating a mechanism by which SUD and PLpro subvert the host’s antiviral defenses [136,137]. The cytokine storm elicited by CoV infection, oxidative stress, and other bacterial infestations potentially activate NF-κB [13].

### 5.2. Suppression of NF-κB Activity

Older macaques infected with SARS-CoV showed increased lung NF-κB activity, leading to a more robust immune response than younger adult macaques. This was accompanied by a significant rise in inflammatory gene expression, primarily regulated by NF-κB [138]. The inhibition of NF-κB could be a viable approach to combat pathogenic SARS-CoV. Targeting NF-κB is challenging due to its intricate involvement in various cellular functions. Agents that block NF-κB lack precision and can disrupt normal cellular balance, potentially leading to adverse side effects such as broadly suppressing the innate immune response [139].

Additionally, viruses could evade NF-κB inhibition by producing proteins that specifically counteract this pathway [139]. Therefore, an alternative strategy could directly target the pathway’s downstream components, such as TNF-α, which NF-κB primarily controls. While TNF-α is crucial in orchestrating the initial inflammatory response, excessive and prolonged production of TNF-α might lead to diminished effectiveness by potentially altering signaling thresholds. Despite numerous other inflammatory factors contributing to the cytokine storm, selectively inhibiting TNF-α has shown clinical effectiveness in various health conditions [140].

**Figure 5:**
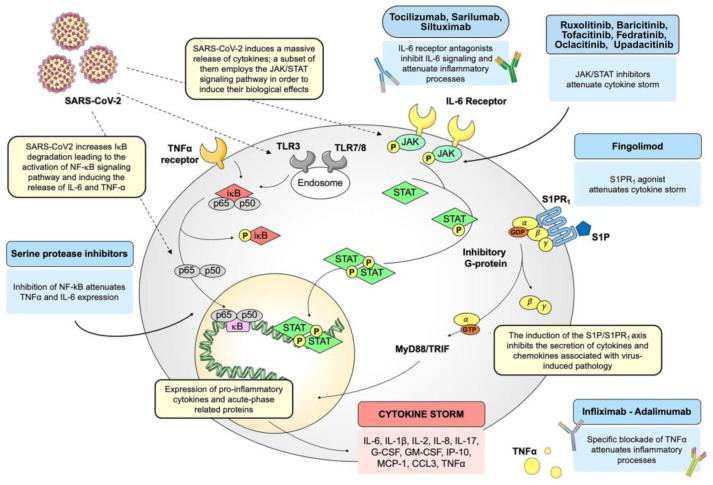
Illustrates a diagrammatic representation of the intracellular signaling pathways activated by SARS-CoV-2 infection. Specific drugs targeting these pathways are repurposed to regulate the excessive release of cytokines induced by the viral infection. CCL3: Chemokine (C-C motif) ligand 3; G-CSF: Granulocyte-colony stimulating factor; GM-CSF: Granulocyte macrophage-colony stimulating factor; IκB: Inhibitor of nuclear factor-κB; JAK: Janus kinase; IL: Interleukin; IP-10: Interferon-γ-induced protein-10; MCP-1: Monocyte chemoattractant protein-1; MyD88: Myeloid differentiation primary response gene 88; NF-κB: Nuclear factor-κB; S1P: Sphingosine-1-phosphate; S1PR1: Sphingosine-1-phosphate receptor 1; STAT: Signal transducer and activator of transcription; TNFα: Tumor necrosis factor α; TLR: Tol-like receptor; TRIF: TIR-domain-containing adapter-inducing IFN-β. (Adapted and updated with permission from Catanzaro et al. [161]).

As a result, medications inhibiting TNF-α, such as infliximab and adalimumab, have proven effective in the treatment of immune-related conditions, including ankylosing spondylitis, inflammatory bowel diseases, psoriasis, and rheumatoid arthritis (RA) [141,142]. Therefore, monoclonal antibodies targeting TNF-α are likely to intensify the inflammatory responses seen in COVID-19, reducing the release of other agents that worsen inflammation. When these antibodies are given to individuals with active RA, it has been shown to quickly reduce a wide range of cytokines (such as IL-6 and IL-1), along with other proteins associated with acute-phase reactions and factors affecting blood vessel permeability [143,144,145]. The exploration of repurposing existing drugs that target precise signal transducers will be examined as potential therapeutic avenues for addressing COVID-19 management, as illustrated in Figure 5.

Additionally, the spike protein of SARS-CoV has been found to enhance the TNF-α-converting enzyme activity that leads to the shedding of the ACE2 receptor [146]. This is a crucial step for the virus to enter cells. Therefore, TNF-α blockers are effective treatments against SARS-CoV infections, as they work in two ways: calming inflammation and hindering viral entry [135]. However, it is important to be cautious about the increased risk of bacterial and fungal infections that may come with anti-TNF-α therapy [147]. Numerous serine protease inhibitors exhibiting chymotrypsin-like specificity, such as DCIC, TPCK, TLCK, BTEE, and APNE, demonstrate the capacity to inhibit proteasome function. In contrast to protease inhibitors exclusively hindering IκB degradation, serine protease inhibitors can obstruct IκB phosphorylation and degradation. However, not all serine protease inhibitors can inhibit NF-κB activation [148,149,150,151].

Moreover, concerning a prospective intervention directed at the NF-κB signaling pathway, inhibitors of serine proteases such as camostat mesylate, nafamostat mesylate, gabexate mesylate, and ulinastatin, frequently employed in the treatment of conditions like pancreatitis, disseminated intravascular coagulation, and as anticoagulants during hemodialysis, have shown efficacy in impeding viral replication and mitigating inflammatory responses across diverse medical contexts, including asthma, chronic allergic pulmonary inflammation, and inflammatory damage to the heart. For example, nafamostat mesylate and gabexate mesylate have been shown to reduce allergen-triggered airway inflammation and the presence of eosinophils in a mouse model of allergic asthma. It also reduces mast cell activation, lowers levels of eosinophils in the lung, and reduces the production of IL-4 and TNF-α driven by *Dermatophagoides pteronyssinus* in the bronchoalveolar lavage fluid [152,153,154,155,156,157,158,159].

Furthermore, gabexate mesylate has demonstrated its ability to hinder the production of TNF-α in human monocytes stimulated by lipopolysaccharide (LPS) and obstruct NF-κB and MAPK activation [160]. Consequently, serine protease inhibitors inhibit complement pathways and exhibit broad-spectrum anti-inflammatory properties, considering their application in managing COVID-19 [161]. However, the precise mechanism by which serine protease inhibitors bring about their anti-inflammatory effects remains unidentified.

**Figure 6:**
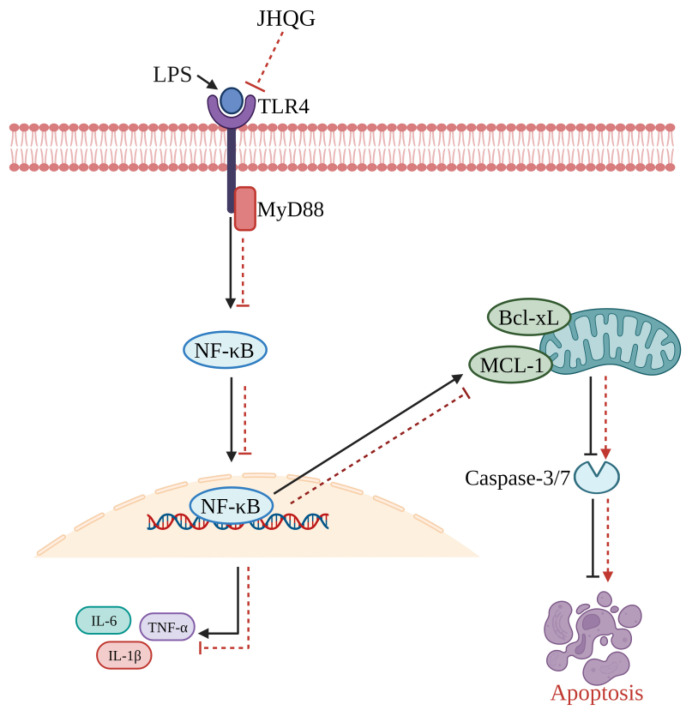
A visual representation of how Jinhua Qinggan granules (JHQG) mitigate acute lung injury (ALI) induced by lipopolysaccharide (LPS) in mice. LPS triggers inflammatory responses through the TLR4/MyD88/NF-κB pathway, leading to excessive production of proinflammatory cytokines (such as TNF-α, IL-1β, and IL-6) and subsequent tissue damage. Furthermore, the activated NF-κB enhances the presence of anti-apoptotic proteins Mcl-1 and Bcl-xL, while inhibiting the activity of caspase 3/7, ultimately prolonging the survival of neutrophils. In contrast, treatment with JHQG suppresses the TLR4/MyD88/NF-κB pathway and facilitates neutrophil apoptosis by reducing Mcl-1 and Bcl-xL and activating caspase 3/7. This demonstrates the protective effect of JHQG in ALI. Bcl-xL: B-cell lymphoma-extra-large; NF-κB: Nuclear factor-κB; IL: Interleukin; TNFα: Tumor necrosis factor α; MyD88: Myeloid differentiation primary response gene 88; TLR: Tol-like receptor; Mcl-1: Myeloid leukemia 1 (Recreated based on Zhu et al. [162]).

Likewise, acute lung injury (ALI) is commonly associated with respiratory viral infections such as influenza and coronaviruses. It carries a significant risk of clinical complications and fatalities. Jinhua Qinggan granules (JHQG) have received approval from the China Food and Drug Administration for treating H1N1 influenza and mild to moderate cases of novel COVID-19. The herbal remedy is derived from traditional Chinese medicine formulations, specifically the Maxingshigan decoction and Yinqiao powder, used to treat respiratory ailments in China for thousands of years [162]. JHQG could enhance the survival of mice with sepsis and reduce lung inflammation caused by LPS (Figure 6). It is achieved by encouraging the natural cell death of neutrophils and inhibiting a specific cellular pathway known as TLR4/MyD88/NF-κB. The results indicate that JHQG could be a valuable herbal medicine for treating ALI triggered by different factors [162].

For effective interventions against COVID-19 complications, three distinct approaches have shown promising results. The first involves the development of an extinguisher, a bioengineered neutrophil construct designed to combat the hyperinflammatory cytokine storm, one of the characteristics of COVID-19 pneumonia. Comprising live neutrophils encapsulating the liposome formulation of NF-κB suppressor MLN4924 and STING inhibitor H-151 (Lip@MH), the extinguisher can uniquely navigate inflamed tissues precisely. Once at the target site, it releases Lip@MH, facilitating the transport of anti-inflammatory agents into macrophages. The dual action effectively inhibits NF-κB and STING-mediated inflammatory pathways, leading to a notable reduction in cytokine production [163]. Remarkably, in animal studies, extinguishers demonstrated selective accumulation at the pneumonia site and tangible mitigation of the cytokine storm through regulating NF-κB/STING signaling pathways, ultimately resulting in improved therapeutic outcomes [163].

Similarly, the Xuebijing formula (XBF) treatment exhibits notable potential in improving lung injury induced by LPS. This treatment modality yielded observable reductions in histopathological alterations, pulmonary alveolar permeability, fibrosis, and apoptosis within lung tissues. In parallel, XBF displayed anti-inflammatory properties, as evidenced by reduced levels of crucial inflammatory markers such as TNF-α, IL-6, and IL-1β. Intriguingly, in cell-based experiments, XBF suppressed the release of inflammatory cytokines and hindered proinflammatory macrophage polarization. Mechanistically, these effects were achieved through the modulation of mitochondrial dynamics and the repression of NLRP3 inflammasome activation, subsequently culminating in the inhibition of the NF-κB and MAPK signaling pathways [134].

Phenoxybenzamine, thimerosal, parthenolide, and auranofin exhibited inhibitory actions on NF-kB-dependent transcription and on forming NF-kB-activating complexes. Auranofin, previously demonstrated to impede SARS-CoV-2 replication [164,165], was assessed for its impact on the NF-kB pathway after 48 h of post-SARS-CoV-2 infection (MOI 0.05) in Calu-3 cells. The investigation aimed to ascertain whether auranofin, alongside parthenolide and ML120B, recognized as NF-kB activation inhibitors, could confer protection against SARS-CoV-2 infection. Vero E6 cells were pretreated with incremental drug dilutions for 2 h, subsequently infected with SARS-CoV-2, and subjected to continuous exposure to these compounds over 48 h. Results revealed that auranofin exhibited inhibitory efficacy against SARS-CoV-2 replication, demonstrating an EC_50_ of 1.2 ± 0.2 mM, whereas parthenolide and ML120B did not manifest similar inhibitory effects [166]. Notably, auranofin and parthenolide interfere with NF-kB by inhibiting IKK kinases [167,168].

The JAK/STAT (Janus kinase/signal transducer and activator of transcription) pathway comprises three main components: receptor activation, JAK/STAT pathway activation, and termination through suppressors of cytokine signaling 3. JAK proteins bind to gp130, leading to tyrosine phosphorylation on gp130, creating docking sites for STAT3. Upon binding to gp130, JAKs phosphorylate STAT3s, inducing their dimerization and subsequent translocation to the nucleus [169]. Several JAK inhibitors, including ruxolitinib, baricitinib, tofacitinib, fedratinib, oclacitinib, and upadacitinib, were authorized by the FDA and European Medicine Association [170,171]. They interact with the signaling molecules of JAK1, JAK2, or TYK2. Ruxolitinib, a JAK1/JAK2 inhibitor, significantly improves pulmonary function in up to ~85% of COVID-19 patients with severe pulmonary disease [171] (Figure 5).

IL-6, primarily produced by monocytes and macrophages, is released in response to cellular stimulation induced by IL-1, TNF-α, and TLR activation during pathogen binding. It targets pivotal cytokines such as IL-6 and inhibits signaling pathways, including JAK, JAK/STAT, and NF-κB, promising approaches to modulate the hyperinflammatory response triggered by SARS-CoV-2 infection [172]. Notably, the clinical efficacy of IL-1 inhibition (anakinra) and IL-6 inhibition (tocilizumab or sarilumab) was studied in COVID-19 patients with respiratory insufficiency and hyperinflammation. The study confirms that IL-1 inhibition significantly reduced mortality in COVID-19 patients compared to IL-6 inhibition [173]. Co-administration of an IL-6 inhibitor (siltuximab) and an IL-6 receptor inhibitor is potent enough to treat COVID-19 patients with acute respiratory distress syndrome [174].

The utilization of curcumin with piperine as adjunctive therapy emerged as a notable approach to managing COVID-19. Patients with severe symptoms who were supplemented with curcumin and piperine showed early symptomatic relief and fewer severe complications compared to the control group. Furthermore, curcumin/piperine therapy could reduce the hospitalization period for patients with moderate to severe symptoms and decrease overall mortality. Integrating oral curcumin with piperine as adjunctive symptomatic therapy in COVID-19 holds promise in improving the burden on healthcare systems and offering a natural, safe option for preventing post-COVID thromboembolic events [175].

## 6. Conclusions

Exploring innate immune signaling is poised to considerably broaden the catalog of viral factors influencing NF-κB activation. Investigating the viral induction of NF-κB-dependent gene expression, including epigenetic modifications, is likely to unveil foundational principles governing the specificity and coordination of NF-κB-mediated transcription and the role of PRRs in virus–host interactions. Examining how viruses manipulate PRRs will illuminate crucial mechanisms in host defense and viral evasion. NF-κB is integral in the initial phases of the host’s response to various pathogens. Consequently, the pathogens have amassed diverse molecules that effectively target nearly every aspect of the NF-κB signaling pathway. These countermeasures from the pathogens specifically uphold a subtle equilibrium between activating and inhibiting the NF-κB pathway, otherwise known as a survival strategy tactic of pathogens. The receptors initiating NF-κB pathway activation are not only stimulated by pathogens but also a range of molecules produced by the host. Unchecked activation of NF-κB is linked to several inflammatory diseases, the development of autoimmune syndromes, and the occurrence of cancers. In COVID-19, NF-κB signaling has been implicated in extra-pulmonary complications and systemic effects. Modulating the immune responses by intervening at the NF-κB activation and IκB degradation levels coupled with TNF-α inhibition can reduce cytokine storm and mitigate the severity of COVID-19. The NF-κB pathway emerges as a promising and pivotal therapeutic target in SARS-CoV-2 infection. As a central player in the immune and inflammatory responses, modulating NF-κB activation could offer a strategic avenue for intervention. By understanding and targeting this pathway, we may nullify the severity of the infection, mitigate cytokine overexpression, and improve the effectiveness of treatments against SARS-CoV-2. Further research and clinical investigations into the intricacies of NF-κB activation and modulation provide the potential to unlock innovative therapeutic strategies for managing and combating the ongoing challenges posed by the COVID-19 pandemic.

## Data Availability

No new data was created in this study. Data sharing does not apply to this article.
